# Invasive lobular carcinoma with metastasis to the pectoralis muscle

**DOI:** 10.1016/j.radcr.2025.03.051

**Published:** 2025-04-05

**Authors:** Odai El-Samawi, Alexander M. Satei, Patricia A. Miller

**Affiliations:** a Department of Radiology, Trinity Health Oakland Hospital, Pontiac, MI, USA; b Department of Radiology, Wayne State University School of Medicine, Detroit, MI, USA; c Department of Radiology, Huron Valley Radiology, Ypsilanti, MI, USA

**Keywords:** Breast imaging, Breast cancer, Invasive lobular carcinoma, Metastatic disease, Pectoralis major muscle

## Abstract

A 61-year-old female presented to our breast clinic for her annual screening mammogram, which revealed an irregular, spiculated mass in the upper outer quadrant of the right breast suspicious for malignancy. Ultrasound imaging identified the mass within the upper outer quadrant of the right breast, as well as another mass within the right major pectoralis muscle, raising concern for metastatic involvement. Magnetic resonance imaging further confirmed an irregular, enhancing mass in the right upper outer breast and an area of mass enhancement in the right pectoralis major muscle. Biopsy of both lesions confirmed invasive lobular carcinoma (ILC). ILC metastasis to the pectoralis muscle is exceedingly rare, with few cases described in the literature. Our case highlights the typical and less common patterns of ILC metastasis, its imaging characteristics, and the implications for treatment and prognosis in such cases.

## Introduction

Breast cancer accounts for the largest proportion of cancer diagnoses in women, with invasive ductal carcinoma (IDC) being the most prevalent subtype, followed by invasive lobular carcinoma (ILC). Approximately 13.1 percent of women will be diagnosed with breast cancer; of these cases, approximately 10% will be ILC [1]. ILC can often be multicentric and bilateral, and represents a diagnostic challenge to diagnose early given its infiltrative growth pattern. In cases of ILC metastasis, pectoral muscle involvement is uncommon, with only a limited number of cases documented in the literature.

## Case report

A 61-year-old female presented to our breast clinic for her annual screening mammogram. The patient reported no symptoms. She had a previous history of left-sided ductal carcinoma *in situ* in 2013, treated with lumpectomy and radiation therapy.

**Fig. 1:**
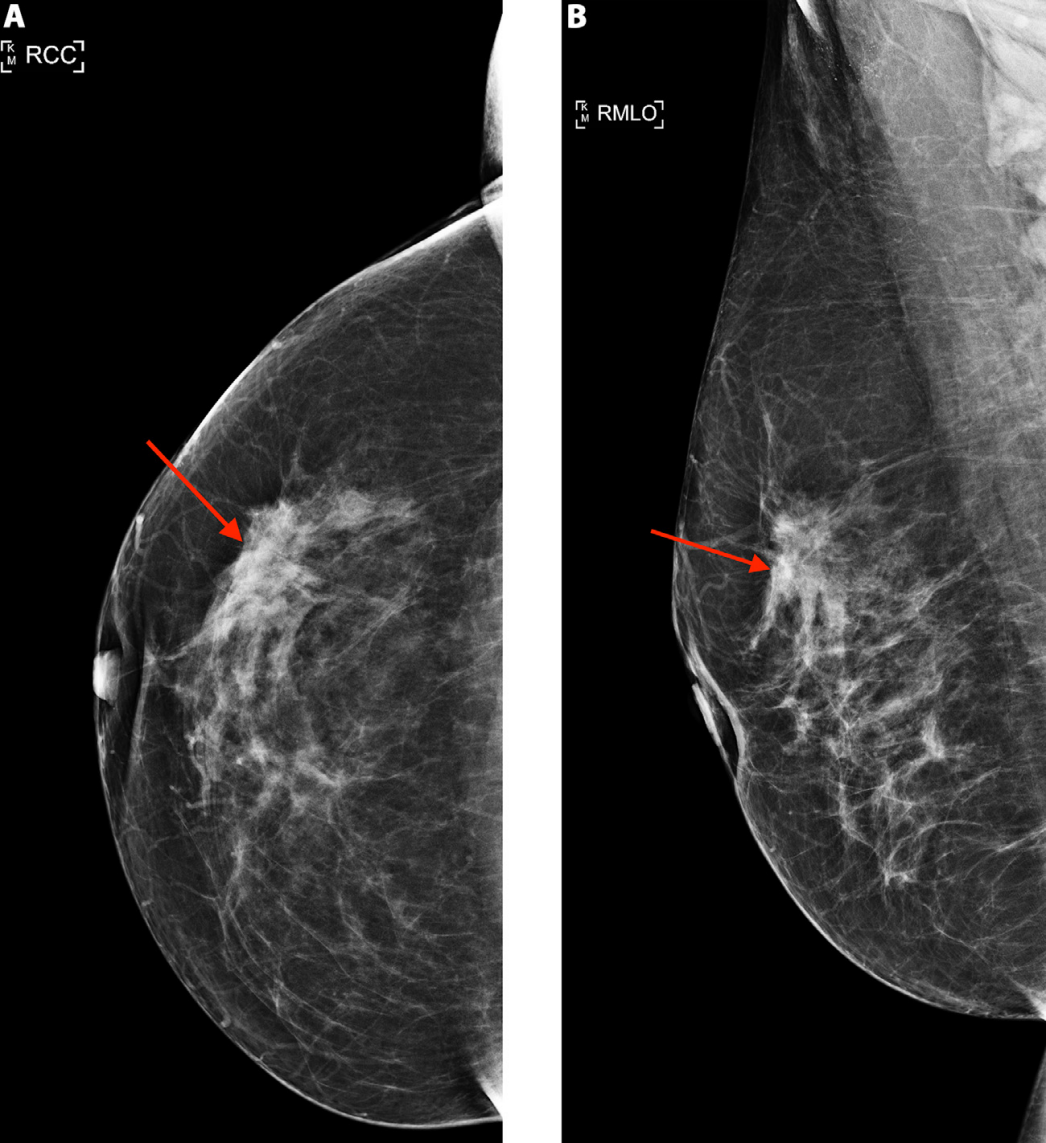
Screening mammogram of the right breast in craniocaudal (A) and right mediolateral oblique (B) views. An irregular spiculated mass measuring 1.8 cm is visualized in the upper outer quadrant of the right breast at middle depth (arrow).

**Fig. 2:**
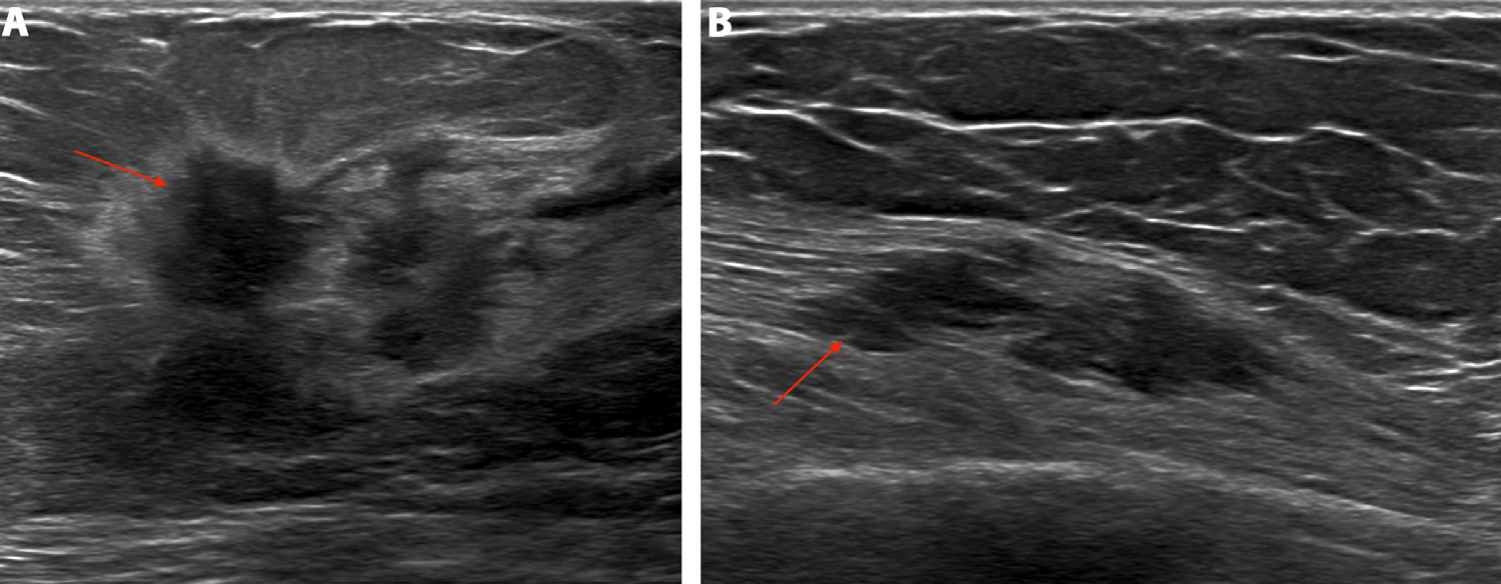
(A) Diagnostic ultrasound of the right breast. At the 10:00 position, with the probe 1 cm from the nipple, an irregularly-shaped hypoechoic mass with spiculated margins is visualized measuring 2.4 × 2.6 × 1.6 cm (arrow). (B) Diagnostic ultrasound of the right breast. At the 11:00 position, with the probe 9 cm from the nipple, a heterogeneously hypoechoic mass within the right pectoralis muscle is visualized measuring 2.9 × 2.6 × 0.8 cm (arrow).

**Fig. 3:**
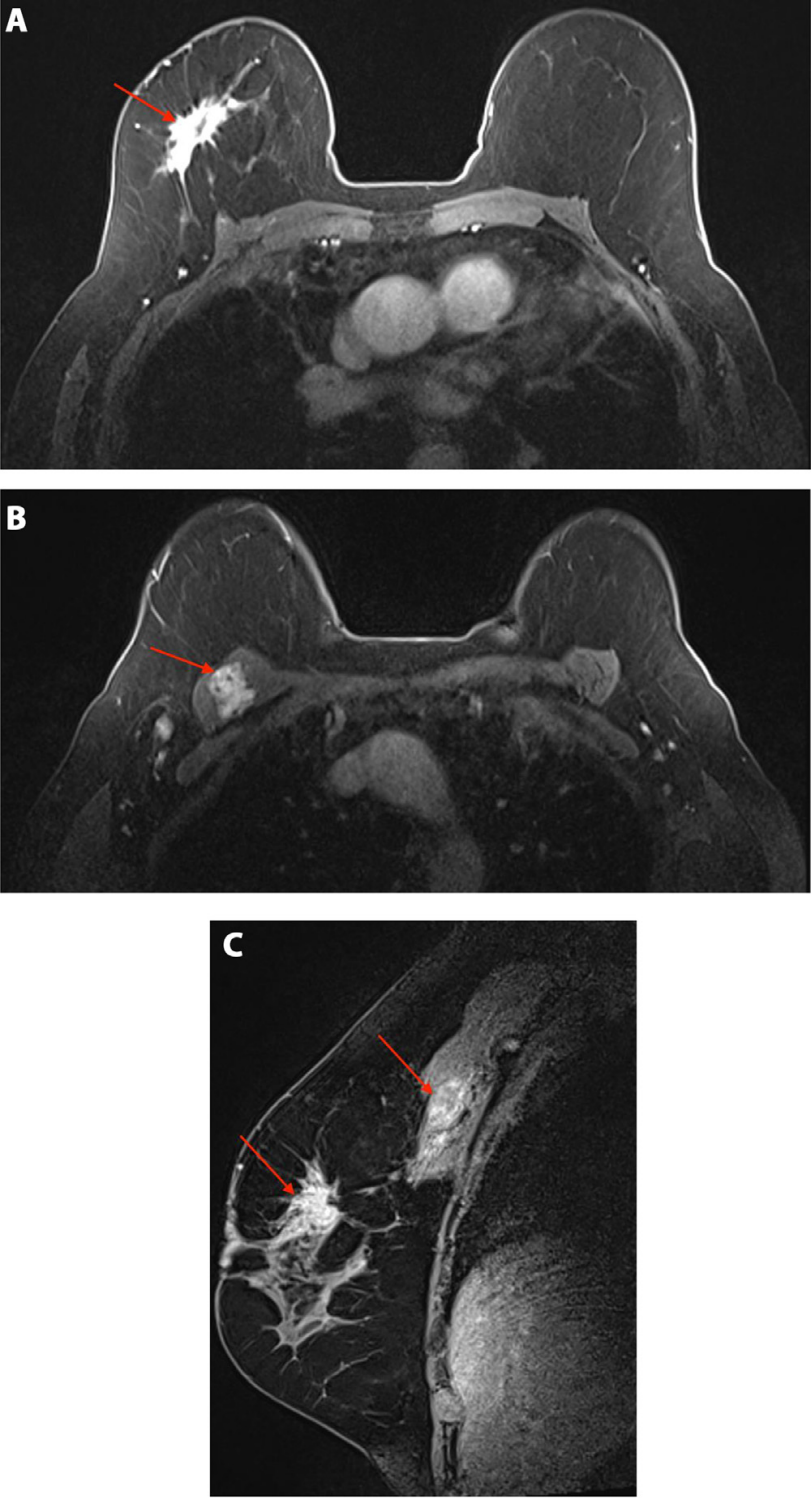
Magnetic resonance imaging of the breasts, contrast enhanced sequences in axial (A, B) and sagittal (C) views. (A) An irregular enhancing mass is visualized at middle depth in the upper outer quadrant of the right breast measuring up to 3.9 cm (arrow). (B) An area of mass enhancement in the right pectoralis major muscle measuring up to 2.1 cm (arrow). (C) Breast and pectoralis major massess (arrows) visualized within the same slice.

**Fig. 4:**
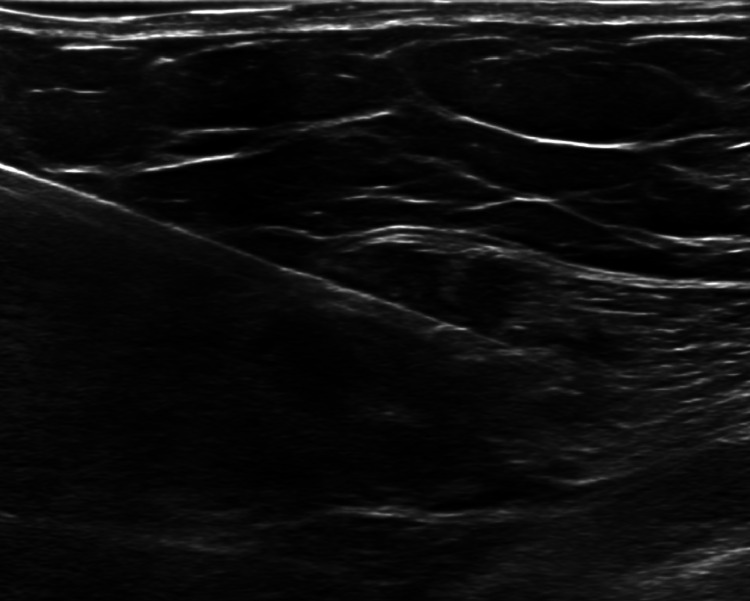
Image from the patient's ultrasound-guided biopsy of the mass within the right pectoralis muscle.

Screening mammography revealed an irregular spiculated mass measuring 1.8 cm in the upper outer quadrant of the right breast at middle depth, 10:00 position (Fig. 1A and B). This mass was evaluated as a Breast Imaging Reporting and Data System (BI-RADS) 4C lesion on the subsequent diagnostic mammogram and ultrasound (Fig. 2A), and was considered highly suspicious for malignancy. Additionally, during the diagnostic ultrasound, a hypoechoic mass was visualized within the right pectoralis major muscle at the 11:00 position, concerning for a focus of metastatic disease (Fig. 2B). Magnetic resonance imaging of the bilateral breasts demonstrated an irregular enhancing mass within the right upper outer breast measuring 3.9 cm (Fig. 3A and B). Additionally, within the right pectoralis major muscle, an area of mass enhancement was visualized measuring 2.1 cm (Fig. 3A and C). Biopsy of the right breast mass performed a week after the diagnostic mammogram and ultrasound was positive for invasive lobular carcinoma. Approximately one month after the initial biopsy, a biopsy of the mass in the right pectoralis major muscle was performed, which was also positive for invasive lobular carcinoma (Fig. 4).

## Discussion

ILC accounts for approximately 10% of all breast cancer diagnoses [1]. Multiple risk factors for ILC have been implicated in the development of ILC, including a family history of breast cancer, prolonged estrogen exposure, a prior history of lobular carcinoma in situ (LCIS), and specific genetic mutations [2]. Mutations in CDH1, which encodes the cadherin gene, are associated with a 50% increased risk of developing ILC. Additionally, BRCA1 and BRCA2 mutations are recognized as risk factors for ILC, with BRCA2 mutations having a higher incidence of the disease. ILC tumors typically are estrogen receptor (ER) and progesterone receptor (PR) positive, with HER2 positivity occurring in 3%-7% of cases [[2], [3], [4]]. The patient described in our case did not have any known genetic mutations; her breast cancer was estrogen receptor positive and HER2 negative.

ILC presents a clinical challenge in both detection and assessment in metastasis, particularly when metastasis involves atypical sites such as the pectoralis muscle. Clinically, ILC may manifest as a palpable area of skin thickening or swelling. As the disease progresses, other findings can include nipple retraction, nipple inversion, and skin tethering [5,6]. However, these findings are nonspecific and can be observed in other types of breast cancer. Approximately 4%-6% of patients with newly diagnosed ILC present with distant metastasis. ILC is more commonly associated with metastasis to the gastrointestinal tract, peritoneum, and retroperitoneum [[7], [8], [9]]. In contrast, involvement of the pectoralis muscle is exceedingly rare, with only a limited number of cases reported in literature [10].

ILC is challenging to detect on imaging due to its tendency to infiltrate through the breasts without forming a distinct mass. Mammography can be limited in its ability to identify ILC, as the tumor typically presents with irregular borders and relatively low density, allowing it to blend in with normal fibroglandular tissue [11]. Approximately 30% of ILC cases are occult on mammography and in the majority of cases the size of the tumor is underestimated [12]. The use of tomosynthesis significantly improves sensitivity for ILC detection compared to mammography alone [13]. On ultrasound, ILC has a nonspecific appearance, commonly presenting as an irregular hypoechoic mass with poorly defined borders. Posterior acoustic shadowing may be seen in over half of cases. Ultrasound also underestimates the size of ILC tumors, and is considered the least sensitive of imaging modalities [12]. MRI is the most sensitive modality for detecting ILC, with a sensitivity of greater than 95%, and is considered the most accurate in estimating tumor extent. MRI typically reveals an irregular spiculated mass with heterogeneous enhancement, with nonmass enhancement being a less frequent appearance [11,14]. Evaluation for bilaterality, multifocality, or metastasis to structures outside of the breast, such as the pectoralis muscle as seen in our case, can also be assessed [15].

In patients with ILC, treatment involves a combination of surgery, chemotherapy, and radiation therapy. Prior to initiation of treatment, a preoperative MR is often obtained to verify the extent of the cancer and to inform the best treatment approach. Surgical excision with clear margins may be performed, however, due to the diffuse nature of ILC, mastectomy is considered especially in cases of multifocal or multicentric disease. Postoperatively, radiation therapy is administered for extensive disease. Neoadjuvant chemoradiation is considered in patients with tumors with advanced stage [16,17]. Selection of hormonal agents is guided by ER/PR/HER2 status. In patients with ER/PR-positive tumors, tamoxifen is utilized for premenopausal women, while an aromatase inhibitor is preferred in postmenopausal women [18,19]. In HER2 positive tumors, trastuzumab is the drug of choice [20]. In cases involving pectoralis muscle invasion, excision of the muscle is performed along with the appropriate surgical and systemic therapies. Ultimately a multidisciplinary approach involving surgeons, oncologists, and radiation therapists is essential to formulate an effective treatment regimen tailored to a patient's clinical situation. Our patient was treated with mastectomy, endocrine therapy utilizing letrozole, an aromatase inhibitor, and adjuvant chemotherapy consisting of doxorubicin and cyclophosphamide with pegfilgrastim support.

Research indicates that the 5-year survival rate for patients with ILC and distant metastasis is approximately 22%. The median overall survival for patients with metastatic ILC, across all receptor subtypes, is approximately 30.2 months [21,22]. Multivariate survival analyses have shown no significant impact of histological subtype on survival outcomes. However, several factors have been identified as contributing to a poorer prognosis, including delayed treatment initiation, negative hormone receptor status, lack of HER2 overexpression, high tumor grade, advanced tumor stage at diagnosis, and metastases to sites beyond bone, such as the liver, peritoneum, or other visceral and nonvisceral organs [23].

## Conclusion

Invasive lobular carcinoma (ILC) is a rare subtype of breast cancer, accounting for approximately 10% of all cases; metastasis to the pectoralis muscle is even more uncommon. Detecting ILC can be challenging due to its subtle presentation on imaging, with MRI being the most sensitive modality for identifying multifocal involvement, including metastasis to the pectoralis muscle. Treatment for metastatic ILC typically involves a multidisciplinary approach, combining surgery, chemotherapy, radiation, and targeted therapies, tailored to the individual patient and the specific characteristics of the tumor. Timely recognition of metastatic disease, particularly pectoralis muscle invasion, along with appropriate radiographic assessment, is essential to ensure proper initiation of treatment and achieving the best possible patient outcomes.

## Patient consent

Informed consent was obtained from the patient for publication of this report and accompanying images.
